# Supplementary material for: Comprehensive short and long read sequencing analysis for the Gaucher and Parkinson’s disease-associated GBA gene
Source: Commun Biol. 2022 Jul 6;5:670. doi: 10.1038/s42003-022-03610-7 (PMC9259685; doi:10.1038/s42003-022-03610-7)
Supplement: Supplementary file 2 — Supplemental Material [file 42003_2022_3610_MOESM2_ESM.pdf]

Supplementary figures

Supplementary Figure 1: Confirmation of the structure of 4 reciprocal recombinant alleles with ONT.

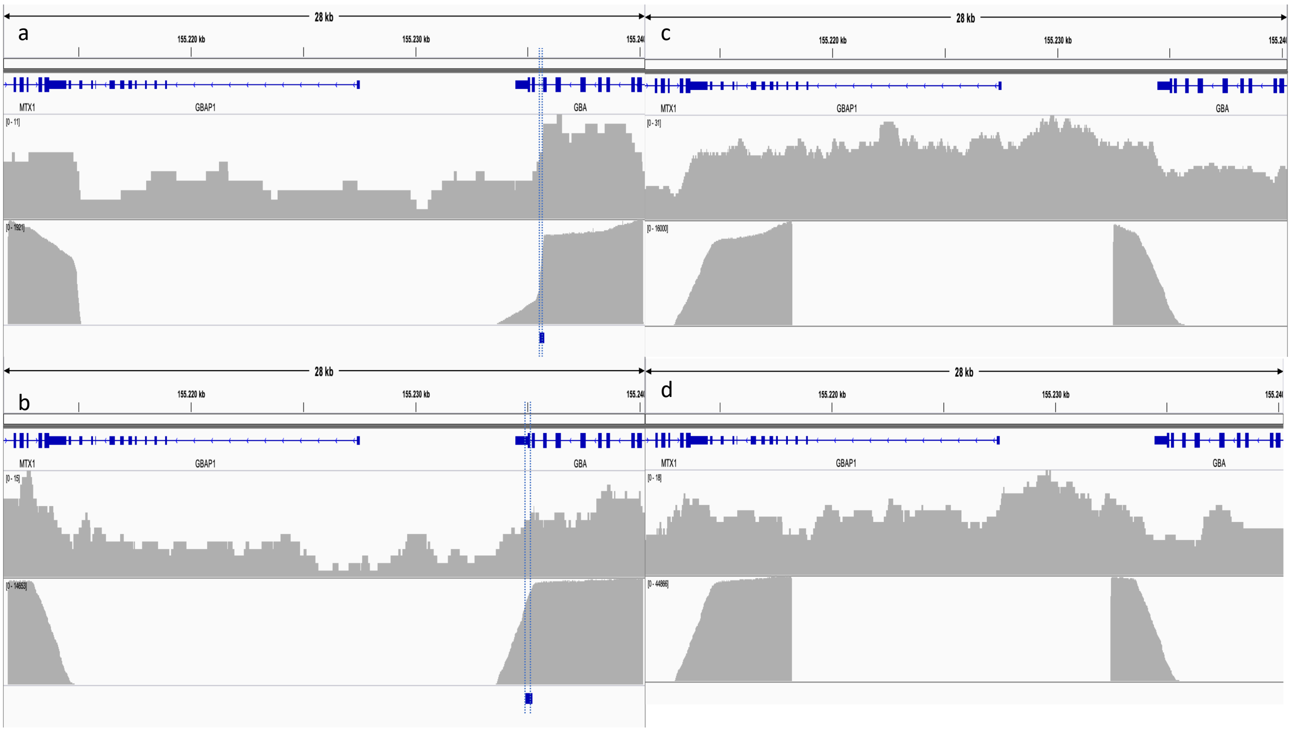

Each panel shows results of one sample: genomic sequencing with adaptive sampling (top), and sequencing of PCR product obtained with primers 2 or 3 (bottom). **a)** Pathogenic CNL allele. **b)** non-pathogenic CNL allele. **c)** CNG allele with a copy number of 5 copies as called by Gauchian 5. **d)** CNG allele with a copy number of 3 copies as predicted by Gauchian.
For the fusion recombinants (A and B), the blue squares and dotted lines at the bottom show the position of the breakpoint predicted with ONT (as described in methods). The breakpoint cannot be refined further as the sequences are identical; see also figure S2. Alignment with LAST.
Genomic region depicted: g.chr1:155,211,770-155,240,380

Supplementary Figure 2: Detection of SNVs in homopolymers with ONT.

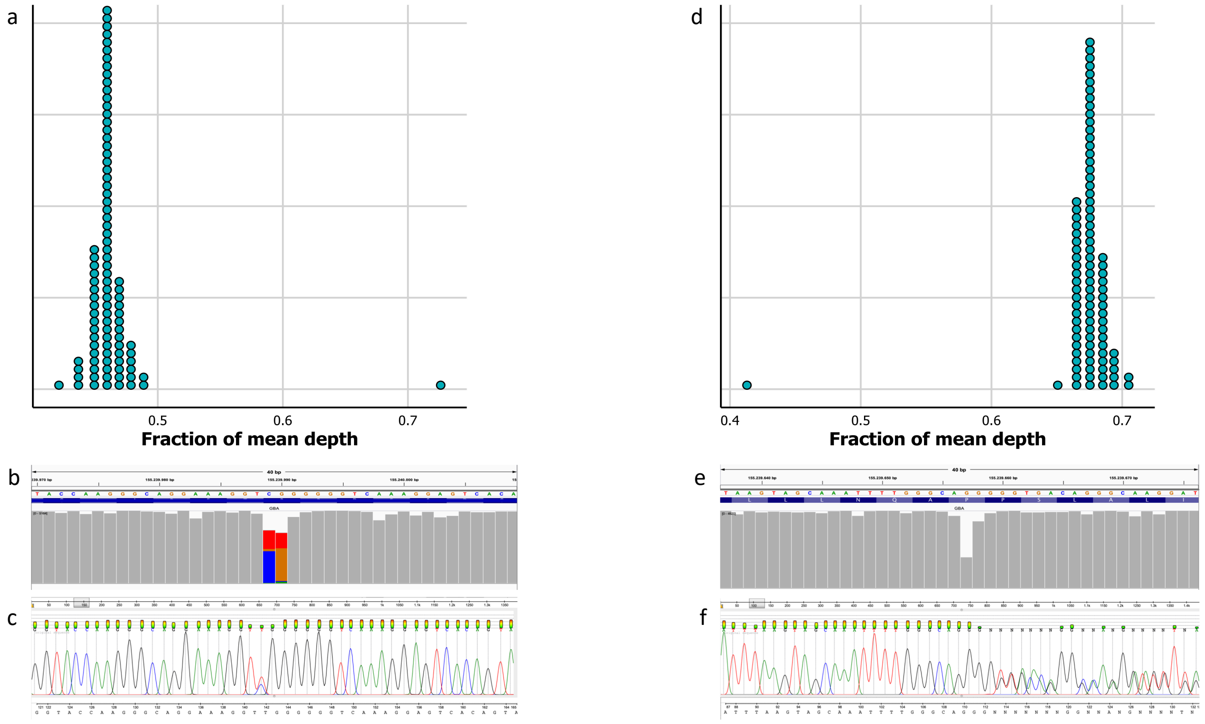

At position chr1:155,239,990 one sample shows an adjusted depth of coverage that is more than 5 median absolute deviations (MAD) higher than the mean of the other samples **(a)**. Inspection of the sequencing data obtained from ONT (primer pair 1) shows that this is probably due to the presence of a single base substitution at position chr1:155,239,989 **(b)**, as confirmed by sanger sequencing **(c)**. Similarly, at position chr1:155,239,657, one sample shows an adjusted depth of coverage that is more than 5 MAD lower than the mean of the other samples, suggesting the deletion of a G in the poly-G. This corresponds to the variant NM_001005742.3:c.413delC **(d)**. Inspection of the sequencing data obtained from ONT (primer pair 1) confirms the drop in depth of coverage **(e)** and Sanger sequencing confirms the presence of a deletion in the poly-G sequence **(f)**.

Supplementary Figure 3: Visual display of two reciprocal fusion recombinant alleles.

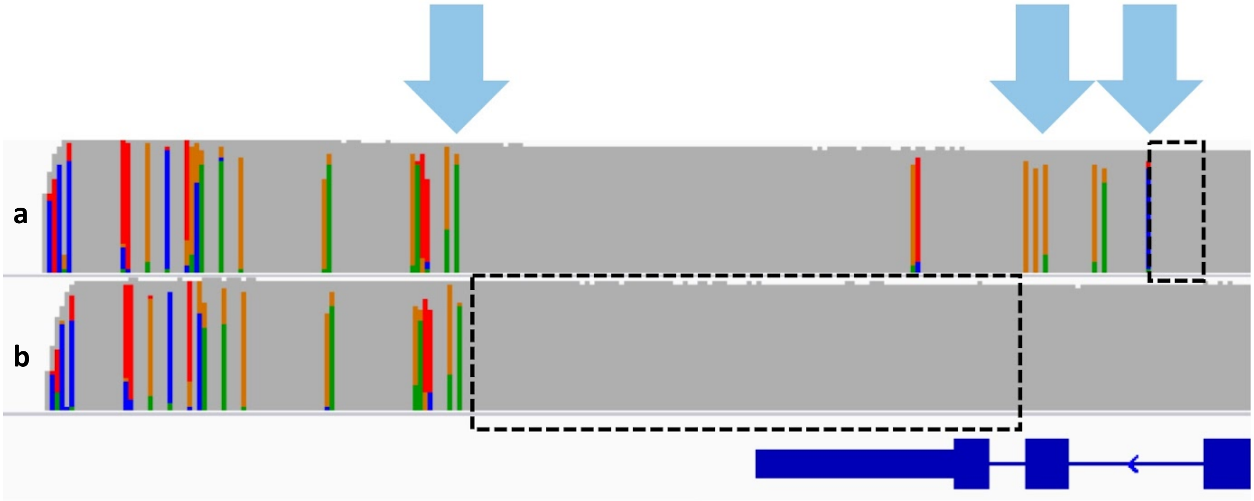

The light blue arrows represent the sentinel positions where *GBA* and *GBAP1* are not homologous. The dotted boxes represent the predicted position of the breakpoints. **a)** breakpoint is between exon 9 and intron 9, so this recombinant allele is predicted to be pathogenic. This is a case of GD, where a pathogenic allele is expected, and indeed had been previously reported independently. **b)** Breakpoint does not affect coding sequence. Exon 11 of *GBA* is 100% homologous to *GBAP1*, so recombinations involving this area do not alter the ORF, so this recombinant is non-pathogenic.

Supplementary Figure 4: IGV screenshot showing the p.L483R variant.

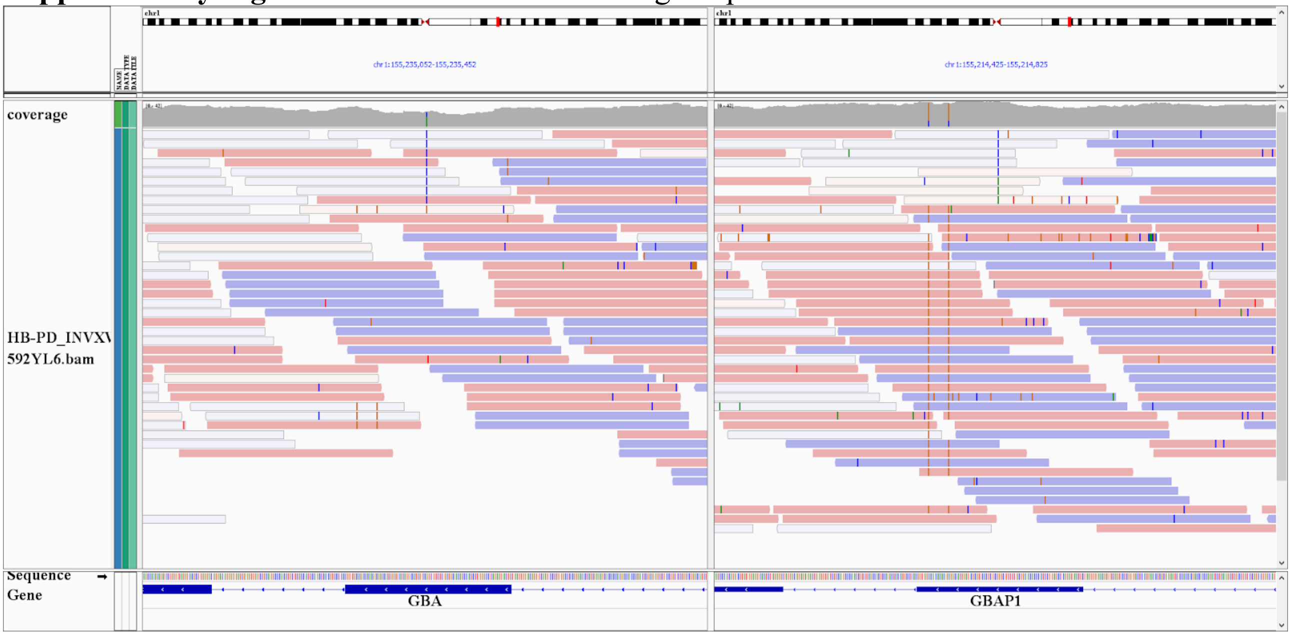

Reads carrying the C variant in the middle are aligned to either *GBA* or *GBAP1*. Many of them have a mapping quality of zero. Hence the variant is missed by BWA-GATK.
Genomic region depicted: g.chr1:155,235,052-155,235,452 (left) and g.chr1:155,214,425-155,214,825 (right)

Supplementary tables

Supplementary Table 1: Results of dPCR. The mean dPCR copy number of 2 probes is given.

Sample	Copy number detected by Gauchian	Copy number (dPCR)	ONT CNV detected
**NA20756**	3	3.18	Duplication
**HG01912**	5	5.15	Duplication
**HG01889**	7	6.71	Duplication
**HG02284**	8	7.86	Duplication
**RAP1**	not analysed	0.98	Fusion
**RAP2**	not analysed	2.06	None

Supplementary Table 2: Phenotypes and genotypes of all samples from the RAPSODI cohort.

	Wild type	Heterozygous carriers*	Homozygous and compound heterozygous carriers*	Total
**PD Patients**	195	36	0	231
**GD Patients**	0	0	33	33
**Healthy individuals**	74	43	0	117
**total**	269	79	33	381

*Including pathogenic structural variants.

Supplementary Table 3: All variants detected in the RAPSODI and QSBB cohorts.

Allele detected	Number of alleles
**p.N409S (NC_000001.11:g.155235843T>G)**	35
**p.L483P (NC_000001.11:g.155235252A>G)**	19
**p.E365K (NC_000001.11:g.155236376C>T)**	15
**p.T408M (NC_000001.11:g.155236246G>A)**	4
**p.R502C (NC_000001.11:g.155235196G>A)**	3
**IVS6-2 (NC_000001.11:155238308T>C)**	2
**p.R301H (NC_000001.11:g.155237438C>T)**	2
**p.84GG (NC_000001.11:g.155240661dup)**	1
**p.D419N (NC_000001.11:g.155235814C>T)**	1
**IVS9+1 (NC_000001.11:g.155235680C>T)**	1
**p.K13R (NC_000001.11:g.155240707T>C)**	1
**p.R301G (NC_000001.11:g.155237439G>C)**	1
**p.T270I (NC_000001.11:g.155237531G>C)**	1
**p.T408M + L104= (NC_000001.11:g.155236246G>A + 155239758C>T)**	1
**p.A357D (NC_000001.11:g.155236399G>T)**	1
**p.D354H (NC_000001.11:g.155236409C>G)**	1
**p.G241R (NC_000001.11:g.155238174C>T)**	1
**p.L519P (NC_000001.11:g.155235050A>G)**	1
**p.P211T (NC_000001.11:g.155238234G>T)**	1
**c.413delC (NC_000001.11:g.155239661del)**	1
**RecNciI(p.A495P + p.Val499= + p.L483P) / reciprocal**	5
**RecNciI(p.A495P + p.Val499= + p.L483P) / non-reciprocal**	1
**RecTL (p.A495P + p.Val499= + p.L483P + p.D448H) + c.1263del / non-reciprocal**	2
**Non-pathogenic reciprocal CN loss recombinants**	4*
**Non-pathogenic reciprocal CN gain recombinants**	7**

These do not include the variants found in the 95 samples previously reported (Salazar et al, 2019)
*In two alleles, a non-pathogenic CNL was detected in cis with p.L444P
**In two samples, a CNG was detected together with c.1263del+RecTL (gene conversion). In two other samples, a duplication recombinant was detected together with the p.L483P variant. These were not phased.

Supplementary Table 4: List of *GBAP1*-like variant calls in Exon 9-11 homology region in 1kGP and AMP-PD cohorts.

sampleID	IsBiallelic	IsCarrier	CN(GBA+GBAP1)	Deletion breakpoint in *GBA*	Recombinant variant allele1	Recombinant variant allele2
**HG00422**	FALSE	TRUE	3	TRUE	RecNciI	
**LB-00540**	FALSE	TRUE	3	TRUE	RecNciI	
**LB-00542**	FALSE	TRUE	3	TRUE	RecNciI	
**LB-00599**	FALSE	TRUE	3	TRUE	RecNciI	
**LB-00695**	FALSE	TRUE	3	TRUE	RecNciI	
**LB-01632**	FALSE	TRUE	3	TRUE	c.1263del+RecTL	
**LB-01652**	FALSE	TRUE	3	FALSE	L483P	
**LB-01755**	FALSE	TRUE	3	TRUE	RecNciI	
**LB-01782**	FALSE	TRUE	3	TRUE	RecNciI	
**LB-01788**	FALSE	TRUE	3	TRUE	RecNciI	
**LB-02286**	FALSE	TRUE	3	TRUE	RecNciI	
**LB-02338**	FALSE	TRUE	3	TRUE	RecNciI	

**LB-02662**	FALSE	TRUE	3	TRUE	c.1263del+RecTL	
**LB-02817**	FALSE	TRUE	3	TRUE	RecNciI	
**HB-PD_INVDX373MWV**	FALSE	TRUE	3	FALSE	L483P	
**HB-PD_INVWN635UXR**	FALSE	TRUE	3	TRUE	RecNciI	
**PD-PDEK306DBC**	FALSE	TRUE	3	FALSE	L483P	
**PD-PDLR687WTH**	FALSE	TRUE	3	TRUE	c.1263del+RecTL	
**HG00119**	FALSE	TRUE	4	None	c.1263del+RecTL	
**NA20815**	FALSE	TRUE	4	None	L483P	
**NA18991**	FALSE	TRUE	4	None	L483P	
**HG02613**	FALSE	TRUE	4	None	A495P	
**HG00115**	FALSE	TRUE	4	None	c.1263del+RecTL	
**HG02439**	FALSE	TRUE	4	None	c.1263del	
**NA20513**	FALSE	TRUE	4	None	L483P	
**HG00310**	FALSE	TRUE	4	None	L483P	
**HG02151**	FALSE	TRUE	4	None	c.1263del	
**HG03967**	FALSE	TRUE	4	None	L483P	
**LB-00009**	FALSE	TRUE	4	None	RecNciI	
**LB-00092**	FALSE	TRUE	4	None	L483P	
**LB-00483**	FALSE	TRUE	4	None	RecNciI	
**LB-00490**	FALSE	TRUE	4	None	L483P	
**LB-00575**	FALSE	TRUE	4	None	c.1263del	
**LB-00668**	FALSE	TRUE	4	None	L483P	
**LB-00734**	FALSE	TRUE	4	None	L483P	
**LB-00760**	FALSE	TRUE	4	None	L483P	
**LB-00906**	FALSE	TRUE	4	None	A495P	
**LB-01188**	FALSE	TRUE	4	None	L483P	
**LB-01223**	FALSE	TRUE	4	None	c.1263del	
**LB-01297**	FALSE	TRUE	4	None	L483P	
**LB-01339**	FALSE	TRUE	4	None	A495P	
**LB-01351**	FALSE	TRUE	4	None	L483P	
**LB-01363**	FALSE	TRUE	4	None	L483P	
**LB-01388**	FALSE	TRUE	4	None	L483P	
**LB-01391**	FALSE	TRUE	4	None	L483P	
**LB-01403**	FALSE	TRUE	4	None	L483P	
**LB-01440**	FALSE	TRUE	4	None	A495P	
**LB-01477**	FALSE	TRUE	4	None	c.1263del	
**LB-01540**	FALSE	TRUE	4	None	D448H	
**LB-01599**	FALSE	TRUE	4	None	L483P	
**LB-01613**	FALSE	TRUE	4	None	D448H	
**LB-01631**	FALSE	TRUE	4	None	RecNciI	
**LB-01696**	FALSE	TRUE	4	None	L483P	
**LB-01752**	FALSE	TRUE	4	None	L483P	

**LB-01803**	FALSE	TRUE	4	None	L483P	
**LB-01894**	FALSE	TRUE	4	None	L483P	
**LB-01922**	FALSE	TRUE	4	None	L483P	
**LB-02197**	FALSE	TRUE	4	None	L483P	
**LB-02483**	FALSE	TRUE	4	None	A495P	
**LB-02676**	FALSE	TRUE	4	None	c.1263del	
**LB-02760**	FALSE	TRUE	4	None	c.1263del	
**LB-02784**	FALSE	TRUE	4	None	L483P	
**LB-02815**	FALSE	TRUE	4	None	c.1263del+RecTL	
**LB-08162**	FALSE	TRUE	4	None	c.1263del	
**LB-06559**	FALSE	TRUE	4	None	A495P	
**LB-07084**	FALSE	TRUE	4	None	D448H	
**LB-07658**	FALSE	TRUE	4	None	A495P	
**BF-1080**	FALSE	TRUE	4	None	A495P	
**HB-PD_INVHN100ZHK**	FALSE	TRUE	4	None	A495P	
**HB-PD_INVKJ120VLF**	FALSE	TRUE	4	None	L483P	
**HB-PD_INVWU757PGL**	FALSE	TRUE	4	None	RecNciI	
**PD-PDCH154ZHF**	FALSE	TRUE	4	None	D448H	
**PD-PDDJ206TP0**	FALSE	TRUE	4	None	RecNciI	
**PD-PDEW749KZU**	FALSE	TRUE	4	None	L483P	
**PD-PDMD724EA9**	FALSE	TRUE	4	None	L483P	
**PD-PDXU108EDQ**	FALSE	TRUE	4	None	L483P	
**PD-PDXU630RLV**	FALSE	TRUE	4	None	L483P	
**PP-3420**	FALSE	TRUE	4	None	L483P	
**PP-3429**	FALSE	TRUE	4	None	A495P	
**PP-3700**	FALSE	TRUE	4	None	L483P	
**PP-41342**	FALSE	TRUE	4	None	L483P	
**PP-60060**	FALSE	TRUE	4	None	L483P	
**HB-PD_INVAV028JED**	FALSE	TRUE	4	None	L483P	
**HB-PD_INVLR599UE9**	FALSE	TRUE	4	None	D448H	
**HB-PD_INVLU007ANL**	FALSE	TRUE	4	None	L483P	
**PD-PDGF687UAV**	FALSE	TRUE	4	None	L483P	
**PP-57787**	FALSE	TRUE	4	None	L483P	

**PP-59343**	FALSE	TRUE	4	None	L483P	
**PP-59926**	FALSE	TRUE	4	None	L483P	
**LB-00239**	FALSE	TRUE	5	None	L483P	
**BF-1016**	FALSE	TRUE	5	None	L483P	
**HB-PD_INVRH572AT6**	FALSE	TRUE	5	None	L483P	
**PP-3307**	FALSE	TRUE	5	None	L483P	
**LB-00823**	FALSE	TRUE	6	None	D448H	
**LB-02672**	TRUE	FALSE	7	None	D448H	L483P
**LB-01354**	FALSE	TRUE	8	None	c.1263del+RecTL	

Supplementary Table 5: Complete list of the number of samples carrying *GBA* variants in population samples.

	1kGP	PD		LBD		
**Variant**	N=2405	case N=2325	control N=1255	case N=2598	control N=1941	mild/severe
**c.115+1G>A**	2	3	1	3	0	severe
**c.1263del**	2	0	0	6	0	severe
**c.1263del+RecTL**	2	1	0	4	0	severe
**p.A348V**	0	0	0	1	0	mild
**p.A495P**	1	3	0	4	2	mild
**p.D438N**	0	0	0	1	0	severe
**p.D448H**	0	1	1	4	1	severe
**p.E365K**	25	91	21	145	30	non-pathogenic (PD risk)
**p.F252I**	0	1	0	0	0	severe
**p.F298L**	0	0	0	1	0	severe
**p.G241R**	0	1	0	4	0	severe
**p.G364R**	0	0	0	1	0	severe
**p.G416S**	0	0	0	1	0	mild
**p.H350R**	0	0	0	1	0	severe
**p.K118N**	0	0	0	1	0	mild
**p.L29fs**	0	3	0	0	1	severe
**p.L483P**	5	14	6	23	0	severe
**p.L483R**	0	1	0	0	0	severe
**p.N227S**	1	0	0	1	0	mild
**p.N409S**	3	128	158	59	19	mild
**p.P305fs**	0	0	0	1	0	severe
**p.R159W**	0	3	0	3	0	severe
**p.R170C**	0	0	0	1	0	severe
**p.R296Q**	0	3	1	3	0	severe
**p.R398X**	0	0	0	1	0	severe
**p.R502C**	2	2	0	8	0	severe
**p.R535C**	0	0	0	1	0	mild
**p.R535H**	0	6	3	4	1	mild
**p.R87W**	0	0	0	1	0	mild
**p.S235P**	0	1	0	0	0	severe
**p.T408M**	9	38	10	65	32	non-pathogenic (PD risk)
**p.V433L**	0	1	0	1	0	severe
**p.V499L**	0	0	0	1	0	mild
**p.W223R**	0	0	0	1	0	severe
**p.W420X**	0	0	0	1	0	unknown
**RecNciI**	1	3	0	13	0	severe

Supplementary Table 6: Analysis of Trios in the 1kGP cohort.

Proband	variant	father	variant	mother	variant
**HG01053**	T408M	HG01051	T408M	HG01052	None
**HG01096**	E365K	HG01094	None	HG01095	E365K
**HG01258**	N409S	HG01256	None	HG01257	N409S
**HG02514**	c.115+1G>A	HG02512	c.115+1G>A	HG02513	None
**NA07348**	E365K	NA07357	None	NA07345	E365K
**HG03909**	T408M	HG03908	T408M	HG03907	None
**NA12335**	T408M	NA12340	None	NA12341	T408M
**HG02615**	A495P	HG02613	A495P	HG02614	None
**HG03669**	None	HG03667	E365K	HG03668	None
**NA10860**	None	NA11992	E365K	NA11993	None
**NA12386**	None	NA12399	E365K	NA12400	None
**NA12818**	None	NA12829	E365K	NA12830	None
**NA12877**	None	NA12889	N409S	NA12890	None
**NA10865**	None	NA11891	None	NA11892	c.115+1G>A
**NA12767**	None	NA12777	None	NA12778	E365K
**NA19151**	None	NA19150	None	NA19149	N227S
**HG00423**	None	HG00421	None	HG00422	RecNciI
**HG01062**	None	HG01060	None	HG01061	T408M
**HG01505**	None	HG01503	None	HG01504	T408M
**HG01508**	None	HG01506	None	HG01507	T408M
**No variant detected in the remaining 582 trios**					

Supplementary Table 7: Cost of ONT analysis of the *GBA* gene per sample (in GBP_

ONT as performed		Multiplexing at 12x96	Flongle
**Longamp polymerase***	4.7		
**AmpureXP magnetic beads**	1.0		
**flow-cell**	7.7	0.7	0.8
**SQK-LSK109**	0.9		
**Barcoding kit**	1.5	2	
**NEBNext companion**	0.4		
**Electrophoresis**	1-2		
**Qubit analysis**	1-2		
**Other consumables****	1		
**Total*****	18.2	11.7	11.3

*This is the cost per sample for 4 PCR reactions: primer pairs 1-3, and the barcoding PCR for primer pair 1 product. If one of the other PCRs also yields a product, indicating reciprocal recombination, this will also require barcoding and sequencing at additional cost for this sample.
**Estimate of costs for nuclease free water, filtered tips, ethanol, agarose electrophoresis, etc.
***This is an estimate at the time of writing (March 2022) and UK list prices, based on using one full MinION flow-cell for 94 samples (plus 2 controls). Assuming 90% of reads are on target, to reach a coverage of 550x for 94 samples requires 512Mb. The theoretical maximum flow cell yield as per Nanopore website is 50Gb. This suggests that costs could be lowered by further optimising the pipeline, for example higher level barcoding by additionally using the ONT kit SQK-PBK004, or using Flongle flow cells (maximum output 2.8 Gb). Finally, re-using MinION flow-cells after flushing is possible. We do not expect the small amount of DNA carried over to confound analysis, but we have not verified this.

Supplementary Table 8: *GBA*/*GBAP1* differentiating sites. Coordinates are in hg38.

chr	*GBA* position (chr1)	*GBA* base	*GBAP1* position	*GBAP1* base
chr 1	155231496	c	155210868	g
chr 1	155231522	a	155210894	g
chr 1	155231557	t	155210929	c
chr 1	155231692	g	155211064	a
chr 1	155231693	c	155211065	t
chr 1	155231701	t	155211073	c
chr 1	155231818	aca	155211190	a
chr 1	155231834	g	155211204	a
chr 1	155231848	a	155211218	c
chr 1	155231857	t	155211227	c
chr 1	155231870	a	155211240	g
chr 1	155231923	t	155211295	a
chr 1	155231936	a	155211308	g
chr 1	155231937	g	155211309	a
chr 1	155231941	a	155211313	g
chr 1	155231942	g	155211314	a
chr 1	155231989	g	155211363	a
chr 1	155232135	t	155211509	a
chr 1	155232231	g	155211605	a
chr 1	155232340	c	155211714	t
chr 1	155232418	a	155211792	g
chr 1	155232440	t	155211814	c
chr 1	155232447	a	155211821	g
chr 1	155232451	a	155211825	g
chr 1	155232540	g	155211914	c
chr 1	155232549	c	155211923	g

**chr 1**	155232550	g	155211924	a
**chr 1**	155232572	t	155211946	c
**chr 1**	155232721	c	155212095	t
**chr 1**	155232724	c	155212098	t
**chr 1**	155232784	a	155212158	g
**chr 1**	155232834	a	155212208	c
**chr 1**	155232892	g	155212266	t
**chr 1**	155232893	a	155212267	t
**chr 1**	155232916	g	155212290	c
**chr 1**	155232919	a	155212293	g
**chr 1**	155232927	g	155212301	a
**chr 1**	155232935	a	155212309	g
**chr 1**	155232982	g	155212356	a
**chr 1**	155233046	a	155212420	g
**chr 1**	155233268	c	155212642	g
**chr 1**	155233287	g	155212661	a
**chr 1**	155233514	a	155212888	g
**chr 1**	155233517	c	155212891	t
**chr 1**	155233521	t	155212895	a
**chr 1**	155233531	g	155212905	a
**chr 1**	155233612	a	155212985	g
**chr 1**	155233639	g	155213012	a
**chr 1**	155234903	c	155214276	t
**chr 1**	155235203	c	155214576	g
**chr 1**	155235217	c	155214590	g
**chr 1**	155235252	a	155214625	g
**chr 1**	155235379	a	155214752	g
**chr 1**	155235412	g	155214785	a

**chr 1**	155235727	c	155215100	g
**chr 1**	155235749	GGGACTGTCGACAAAGTTACGCACCCAATTGGGTCCTCCTTCGGGGTTCAGGGCAA	155215122	g
**chr 1**	155235918	a	155215236	g
**chr 1**	155236097	g	155215415	c
**chr 1**	155236102	t	155215420	c
**chr 1**	155236129	t	155215447	c
**chr 1**	155236145	g	155215463	c
**chr 1**	155236175	g	155215493	a
**chr 1**	155236190	a	155215508	g
**chr 1**	155237982	t	155216705	c
**chr 1**	155237989	g	155216712	a
**chr 1**	155237990	c	155216713	t
**chr 1**	155237996	g	155216719	t
**chr 1**	155237997	c	155216720	t
**chr 1**	155238055	t	155216778	c
**chr 1**	155238088	t	155216811	c
**chr 1**	155238092	t	155216815	g
**chr 1**	155238141	a	155216864	t
**chr 1**	155238174	c	155216897	t
**chr 1**	155238192	a	155216915	g
**chr 1**	155238206	a	155216929	c
**chr 1**	155238214	a	155216937	c
**chr 1**	155238630	g	155217353	a
**chr 1**	155238754	g	155217477	a
**chr 1**	155238872	ag	155217595	t
**chr 1**	155238882	a	155217604	g
**chr 1**	155238929	a	155217651	t
**chr 1**	155238930	g	155217652	t

Supplementary Table 9: *GBA* variants targeted by Gauchian. Coordinates are in hg38.

chr	position	ref	alt	variant_name
**chr1**	155235002	C	T	p.R535H
**chr1**	155235003	G	A	p.R535C
**chr1**	155235007	C	T	p.W533X
**chr1**	155235057	C	T	p.G517S
**chr1**	155235101	C	T	c.1506-1G>A
**chr1**	155235196	G	A	p.R502C
**chr1**	155235197	G	C	p.N501K
**chr1**	155235205	C	G	p.V499L
**chr1**	155235217	C	G	p.A495P
**chr1**	155235241	C	T	p.A487T
**chr1**	155235252	A	C	p.L483R
**chr1**	155235252	A	G	p.L483P
**chr1**	155235303	A	C	p.I466S
**chr1**	155235679	A	G	c.1388+2T>C
**chr1**	155235708	G	C	p.P454R
**chr1**	155235712	G	A	p.Q453X
**chr1**	155235721	A	T	p.F450I
**chr1**	155235726	T	A	p.D448V
**chr1**	155235727	C	G	p.D448H
**chr1**	155235749	GGGACTGTCGACAAAGTTACGCACCCAAT TGGGTCCTCCTTCGGGGTTCAGGGCAA	G	p.L422fs/c.1263del
**chr1**	155235750	G	A	p.P440L
**chr1**	155235757	C	T	p.D438N
**chr1**	155235760	C	A	p.V437F
**chr1**	155235772	C	A	p.V433L
**chr1**	155235780	G	A	p.P430L
**chr1**	155235790	C	A	p.E427X
**chr1**	155235798	A	G	p.L424P
**chr1**	155235810	C	T	p.W420X
**chr1**	155235819	C	T	p.W417X
**chr1**	155235823	C	T	p.G416S
**chr1**	155235829	C	A	p.V414L
**chr1**	155235831	T	G	p.H413P
**chr1**	155235841	G	C	p.L410V

**chr1**	155235843	T	C	p.N409S
**chr1**	155235843	T	G	p.N409T
**chr1**	155236255	C	G	p.S405T
**chr1**	155236261	C	G	p.S403T
**chr1**	155236277	G	A	p.R398X
**chr1**	155236285	G	A	p.S395F
**chr1**	155236292	G	C	p.L393V
**chr1**	155236295	G	A	p.R392W
**chr1**	155236295	G	C	p.R392G
**chr1**	155236298	C	G	p.V391L
**chr1**	155236318	G	A	p.S384F
**chr1**	155236328	A	C	p.C381G
**chr1**	155236379	C	T	p.G364R
**chr1**	155236384	G	A	p.T362I
**chr1**	155236416	C	A	p.W351C
**chr1**	155236417	C	G	p.W351S
**chr1**	155236420	T	C	p.H350R
**chr1**	155236426	G	A	p.A348V
**chr1**	155236439	CA	C	p.K342_Y343insX
**chr1**	155237099	G	T	c.999+242C>A
**chr1**	155237357	G	A	p.P328L
**chr1**	155237370	G	A	p.R324C
**chr1**	155237394	G	A	p.R316C
**chr1**	155237411	C	T	p.S310N
**chr1**	155237412	T	C	p.S310G
**chr1**	155237425	AG	A	p.P305fs
**chr1**	155237427	G	C	p.P305A
**chr1**	155237444	A	G	p.I299T
**chr1**	155237446	G	T	p.F298L
**chr1**	155237453	C	T	p.R296Q
**chr1**	155237454	G	A	p.R296X
**chr1**	155237470	G	T	p.F290L
**chr1**	155237474	C	G	p.G289A
**chr1**	155237480	C	A	p.C287F

**chr1**	155237576	A	T	p.F255Y
**chr1**	155237577	A	C	p.F255V
**chr1**	155237579	C	G	c.762-1G>C
**chr1**	155238141	A	T	p.F252I
**chr1**	155238174	C	T	p.G241R
**chr1**	155238186	T	C	p.K237E
**chr1**	155238192	A	G	p.S235P
**chr1**	155238194	C	T	p.G234E
**chr1**	155238214	A	C	p.N227K
**chr1**	155238214	AT	CC	p.N227R
**chr1**	155238215	T	C	p.N227S
**chr1**	155238228	A	G	p.W223R
**chr1**	155238234	G	T	p.P221T
**chr1**	155238242	C	T	p.W218X
**chr1**	155238264	CG	C	p.V211fs
**chr1**	155238270	G	A	p.R209C
**chr1**	155238298	CAG	C	p.L199fs
**chr1**	155238519	T	G	p.K196Q
**chr1**	155238525	T	A	p.K194X
**chr1**	155238547	GA	G	p.F186fs
**chr1**	155238571	AG	A	p.P178fs
**chr1**	155238579	C	T	p.D176N
**chr1**	155238584	T	C	p.Y174C
**chr1**	155238596	C	A	p.R170L
**chr1**	155238596	C	T	p.R170H
**chr1**	155238597	G	A	p.R170C
**chr1**	155238608	T	A	p.D166V
**chr1**	155238617	GC	G	p.A163fs
**chr1**	155238624	G	A	p.P161S
**chr1**	155238629	C	T	p.R159Q
**chr1**	155238630	G	A	p.R159W
**chr1**	155239639	A	C	p.L144R
**chr1**	155239655	C	G	p.A139P
**chr1**	155239665	CAG	C	p.L135fs

**chr1**	155239716	C	G	p.K118N
**chr1**	155239736	G	A	p.Q112X
**chr1**	155239934	G	A	p.R87W
**chr1**	155239939	C	T	p.G85E
**chr1**	155239968	GGTA	G	p.T75del
**chr1**	155239989	C	CG	p.T69fs
**chr1**	155240033	C	A	p.V54L
**chr1**	155240629	C	T	c.115+1G>A
**chr1**	155240637	C	T	p.W36X
**chr1**	155240651	G	GC	p.Q32fs
**chr1**	155240660	G	GC	p.L29fs
**chr1**	155240671	AG	A	p.L25fs
**p.L483P + p.A495P + p.Val499=**				RecNciI
**RecNciI + p.D448H**				Rec.TL
**RecNciI + p.D448H + c.1263del**				c.1263del+RecTL

Supplementary Table 10: PCR conditions for primer pair 1.

**Reagents**	**Volume**		**°C**	**Time**	**cycles**
**Template DNA (200ng)**	x µL		94	30sec	1
**Nuclease free water**	19µL - x		94	15sec	35
**Mg2+**	2µL		65	15sec	
**Forward primer**	2µL		65	6min	
**Reverse primer**	2µL		65	10min	1
**LongAmp Taq 2x polymerase mix (NEB)**	25µL		4	hold	
**Primer pair A***	Forward	5′- TTTCTGTTGGTGCTGATATTGCTCCTAAAGTTGTCACCCATACATG -3′			
	Reverse	5′- ACTTGCCTGTCGCTCTATCTTCCCAACCTTTCTTCCTTCTTCTCAA - 3′			

*primers have been edited to contain the ONT barcode adapter sequence.

Supplementary Table 11: PCR conditions for primers pairs 2 and 3.

Reagents	Volume	°C	Time	cycles
**Template DNA (100ng)**	x µL	95	3min	1
**Nuclease free water**	24.5µL - x	95	15sec	12
		62	15sec	
**Barcode mix**	0.5µL	65	10min	
		65	10min	1
**LongAmp Taq 2x polymerase mix (NEB)**	25µL	4	hold	
**Primer pair B***	Forward	5′-TTTCTGTTGGTGCTGATATTGCATGTGTCCATTCTCCATGTCTTCA-3′		
	Reverse	5′-ACTTGCCTGTCGCTCTATCTTCAGCCTTCCTTCCTTCCCTGCAT-3′		
**Primer pair C***	Forward	5′-TTTCTGTTGGTGCTGATATTGCGTGTCCGTTCTCCACATCCTTG-3′		
	Reverse	5′-ACTTGCCTGTCGCTCTATCTTCCCAACCTTTCTTCCTTCTTCTCAA-3′		

*primers have been edited to contain the ONT barcode adapter sequence.
