# Supplementary material for: Comprehensive short and long read sequencing analysis for the Gaucher and Parkinson’s disease-associated GBA gene
Source: Commun Biol. 2022 Jul 6;5:670. doi: 10.1038/s42003-022-03610-7 (PMC9259685; doi:10.1038/s42003-022-03610-7)
Supplement: Supplementary file 3 — Reporting Summary [file 42003_2022_3610_MOESM3_ESM.pdf]

nature portfolio

Corresponding author(s): prof. Christos Proukakis

Last updated by author(s): Jan 7, 2022

Reporting Summary

Nature Portfolio wishes to improve the reproducibility of the work that we publish. This form provides structure for consistency and transparency in reporting. For further information on Nature Portfolio policies, see our Editorial Policies and the Editorial Policy Checklist.

Statistics

For all statistical analyses, confirm that the following items are present in the figure legend, table legend, main text, or Methods section.

n/a	Confirmed	
☐	☒	The exact sample size (*n*) for each experimental group/condition, given as a discrete number and unit of measurement
☒	☐	A statement on whether measurements were taken from distinct samples or whether the same sample was measured repeatedly
☐	☒	The statistical test(s) used AND whether they are one- or two-sided<br>*Only common tests should be described solely by name; describe more complex techniques in the Methods section.*
☐	☒	A description of all covariates tested
☐	☒	A description of any assumptions or corrections, such as tests of normality and adjustment for multiple comparisons
☐	☒	A full description of the statistical parameters including central tendency (e.g. means) or other basic estimates (e.g. regression coefficient) AND variation (e.g. standard deviation) or associated estimates of uncertainty (e.g. confidence intervals)
☐	☒	For null hypothesis testing, the test statistic (e.g. *F*, *t*, *r*) with confidence intervals, effect sizes, degrees of freedom and *P* value noted<br>*Give P values as exact values whenever suitable.*
☒	☐	For Bayesian analysis, information on the choice of priors and Markov chain Monte Carlo settings
☒	☐	For hierarchical and complex designs, identification of the appropriate level for tests and full reporting of outcomes
☒	☐	Estimates of effect sizes (e.g. Cohen's *d*, Pearson's *r*), indicating how they were calculated

Our web collection on statistics for biologists contains articles on many of the points above.

Software and code

Policy information about availability of computer code

Data collection	Gauchian and the pipeline for analysing nanopore data were developed by our groups. Both are available on the relevant Github pages, as indicated in the text. Other software used is: MinION (version 20.10.3), Guppy (version 4.2.2), NGMLR (version 0.2.7), Clair (version 2.1.1), Whatshap (version 1.0), Samtools (version 1.10), Bedtools (version 2.29.1), Tabix (version 1.7-2), LAST (Version 1243).
Data analysis	R (version 4.0.5)

For manuscripts utilizing custom algorithms or software that are central to the research but not yet described in published literature, software must be made available to editors and reviewers. We strongly encourage code deposition in a community repository (e.g. GitHub). See the Nature Portfolio guidelines for submitting code & software for further information.

Data

Policy information about availability of data

All manuscripts must include a data availability statement. This statement should provide the following information, where applicable:

- Accession codes, unique identifiers, or web links for publicly available datasets
- A description of any restrictions on data availability
- For clinical datasets or third party data, please ensure that the statement adheres to our policy

Gauchian can be downloaded from: https://github.com/Illumina/Gauchian
ONT and UNCALLED scripts used can be downloaded at https://github.com/marcotoffoli
Individual-level genome sequence data for the PD patients, LBD patients, and neurologically healthy controls are available at AMP-PD (https://amp-pd.org).
The datasets of DNA from QSBB brain samples and NHGRI samples generated during this study (Illumina WGS and targeted ONT sequencing) will be made available on the European Nucleotide Archive (https://www.ebi.ac.uk/ena/browser/home), accession number PRJEB48317. The datasets only include read alignments to

GBA/GBAP1 regions (other regions of the genome have been removed or masked) to minimize the amount of genetic information made available for public access.
The datasets of DNA from PPMI samples generated during this study (targeted ONT sequencing) will be made available on the PPMI repository (https://www.ppmi-info.org/).
ONT sequencing data on living individuals are not available due to consent / IRB restrictions.
1k GP project https://www.ncbi.nlm.nih.gov/bioproject/PRJEB31736
RAPSODI, https://rapsodistudy.com
OMIM, http://www.omim.org/
The NCBI reference sequence for GBA on which the numbering of exons is based is NM_000157.4.

Field-specific reporting

Please select the one below that is the best fit for your research. If you are not sure, read the appropriate sections before making your selection.

☒ Life sciences ☐ Behavioural & social sciences ☐ Ecological, evolutionary & environmental sciences

For a reference copy of the document with all sections, see nature.com/documents/nr-reporting-summary-flat.pdf

Life sciences study design

All studies must disclose on these points even when the disclosure is negative.

Sample size	This paper reports the validation of two sequencing tools and then describes the findings in the publicly and non-publicly available cohorts we applied this software to. All samples available were analysed, and no sample size calculation was carried out.
Data exclusions	No data were excluded from analysis. For some specific analysis regarding prevalence of GBA variants, samples carrying the variant p.N409S were excluded as these were enriched in the AMP-PD cohort.
Replication	We cross-validated findings with both Gauchian and ONT on 43 samples. We confirmed copy number estimation with digital PCR on 4 samples.
Randomization	No randomisation was required for this work.
Blinding	The cross-validation on the 42 samples with both Gauchian and ONT was carried out without prior sharing of results. No other blinding procedures were required for this work.

Reporting for specific materials, systems and methods

We require information from authors about some types of materials, experimental systems and methods used in many studies. Here, indicate whether each material, system or method listed is relevant to your study. If you are not sure if a list item applies to your research, read the appropriate section before selecting a response.

Materials & experimental systems

n/a	Involved in the study	
☒	☐	Antibodies
☒	☐	Eukaryotic cell lines
☒	☐	Palaeontology and archaeology
☒	☐	Animals and other organisms
☐	☒	Human research participants
☐	☒	Clinical data
☒	☐	Dual use research of concern

Methods

n/a	Involved in the study	
☒	☐	ChIP-seq
☒	☐	Flow cytometry
☒	☐	MRI-based neuroimaging

Human research participants

Policy information about studies involving human research participants

Population characteristics	RAPSODI and Queen Square Brain Bank cohorts: 397 participants (247 Parkinson patients, 33 Gaucher disease patients, 117 healthy controls).<br>AMP-PD cohort: 8199 samples analysed (2325 Parkinson patients, 2598 dementia with lewy bodies patients, 3196 controls)<br>1000 genome project cohort: 2504 participants
Recruitment	RAPSODI participants were recruited through the online RAPSODI portal.<br>Queen Square Brain Bank samples are post-mortem samples, obtained from the biobank<br>AMP-PD and 1000 genome project data are publicly available and were downloaded from the corresponding platforms.
Ethics oversight	Ethics approval was provided by the National Research Ethics Service London—Hampstead Ethics Committee for RAPSODI,

Ethics oversight	NRES Committee central—London for QSBB samples, and UCL Ethics Committee for PPMI samples. All participants provided informed consent.

Note that full information on the approval of the study protocol must also be provided in the manuscript.

Clinical data

Policy information about clinical studies

All manuscripts should comply with the ICMJE guidelines for publication of clinical research and a completed CONSORT checklist must be included with all submissions.

Clinical trial registration	Not applicable
Study protocol	This is not a clinical trial
Data collection	RAPSODI samples were collected between January 2018 and May 2021 and were analysed at our laboratory at the Department of Clinical and Movement Neurosciences, Institute of Neurology, University College London.<br>Remaining data were obtained from publicly available repositories.
Outcomes	This is an observational study and not a clinical trial, and no outcomes were pre-defined.
